# Organic/Inorganic Metal Halide Perovskite Optoelectronic Devices beyond Solar Cells

**DOI:** 10.1002/advs.201700780

**Published:** 2018-03-06

**Authors:** Jiachen Sun, Jiang Wu, Xin Tong, Feng Lin, Yanan Wang, Zhiming M. Wang

**Affiliations:** ^1^ Institute of Fundamental and Frontier Science University of Electronic Science and Technology of China Chengdu 610054 P. R. China; ^2^ Department of Electronic and Electrical Engineering University College London Torrington Place London WC1E 7JE UK

**Keywords:** lasers, light emitting diodes, organic/inorganic hybrid perovskites, photodetectors

## Abstract

Investigations of organic–inorganic metal halide perovskite materials have attracted extensive attention due to their excellent properties including bandgap tunability, long charge diffusion length, and outstanding optoelectronic merits. Organic–inorganic metal halide perovskites are demonstrated to be promising materials in a variety of optoelectronic applications including photodetection, energy harvesting, and light‐emitting devices. As perovskite solar cells are well studied in literature, here, the recent developments of organic–inorganic metal halide perovskite materials in optoelectronic devices beyond solar cells are summarized. The preparation of organic–inorganic metal halide perovskite films is introduced. Applications of organic–inorganic metal halide perovskite materials in light‐emitting diodes, photodetectors, and lasers are then highlighted. Finally, the recent advances in these optoelectronic applications based on organic–inorganic metal halide materials are summarized and the future perspectives are discussed.

## 1 Introduction

**Figure 1:**
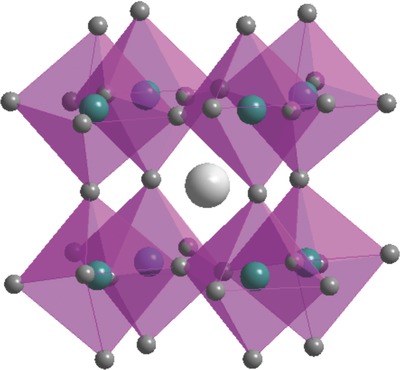
The perovskite structure with a typical formula of ABM_3_. Reproduced with permission.^1^ Copyright 2017, American Chemical Society.

The exploration of organic–inorganic metal halide perovskite materials has been lasted for half century before they have been intensively researched in recent years. Initially, a perovskite material is only referred to as a calcium titanium oxide mineral (CaTiO_3_). Later, the perovskite also represents materials with the same crystal structure with CaTiO_3_. The typical organic–inorganic metal halide perovskite materials can be described as ABX_3_ in which A represents an organic cation, B is a metal cation, and X represents a halide anion such as I^−^, Cl^−^, and Br^−^. In this structure, A cations are surrounded by [BX_6_]^4−^ octahedral, as shown in **Figure** **1**.^1^ Such organic–inorganic hybrid perovskites have been well studied for decades. At the turn of the century, there were many researches focusing on their crystal structures,^2^, ^3^, ^4^, ^5^, ^6^, ^7^, ^8^, ^9^, ^10^, ^11^, ^12^, ^13^, ^14^, ^15^, ^16^, ^17^ optical properties,^3^, ^7^, ^8^, ^11^, ^12^, ^13^, ^15^, ^18^, ^19^, ^20^, ^21^ thermal properties,^3^, ^12^, ^22^ and ferroelectric properties.^6^, ^23^, ^24^, ^25^, ^26^ It is shown that the organic–inorganic hybrid perovskite materials possess merits in magnetism,^27^, ^28^ ferroelectricity, 2D conductivity, and optoelectronics. In order to understand the fundamental properties of these materials, many characterization methods have been applied, which in turn has led to fabrication of these materials into devices. Schmid and co‐workers fabricated an organic–inorganic perovskite light emitting diode (LED) and achieved an external quantum efficiency of 4%.^29^ Later, Mitzi and co‐workers also developed an organic–inorganic perovskite field effect transistor (FET) and found that organic–inorganic hybrid materials were very promising to be a channel material,^10^ which introduced a way for further application of electronic devices.

Among various organic–inorganic metal halide perovskite materials, the ABX_3_ [A = CH_3_NH_3_^+^ (MA^+^), B = metal anion (Pb^2+^, Sn^2+^), X = I^−^, Cl^−^ and Br^−^] organometal perovskite has been emerging as a star material in optoelectronics. The research of this kind of organic–inorganic hybrid perovskite materials can be dated back to the middle of last century.^30^, ^31^ In 1992, Onodayamamuro et al. studied the dielectric properties of MAPbI_3_ and MAPbBr_3_ during their phase transition.^32^, ^33^ Others also studied phase transition in MAPbX_3_ materials through nuclear magnetic resonance and nuclear quadrupole resonance.^34^ In order to study the distortion of the octahedron of halogens through the phase transitions, five types of octahedral deformation were determined by extended X‐ray absorption fine structure.^35^ Raman scattering also showed the evidence of phase transition and cations motions of octahedral distortion in hybrid perovskite materials.^6^, ^36^ All of these studies pointed out that there are three phases in MAPbX_3_: orthorhombic (low temperature phase), tetragonal (intermediate phase), and cubic (high temperature phase).

Those explorations and attempts have provided good understanding of the material properties and proved that organic–inorganic hybrid perovskites are promising to be utilized in optoelectronic devices. However, only until 2009, the first photoelectron conversion device was achieved by Kojima et al.^37^ In this research, the organometal, trihalide perovskite MAPbI_3_ was used as a photo sensitizer in dye‐sensitized solar cells (DSSCs). Although these solar cells only achieved a power conversion efficiency (PCE) of 3.8%, this work has triggered intensive researches in organic–inorganic hybrid perovskite materials.

In 2011, CH_3_NH_3_PbI_3_ nanocrystals were employed on a nanocrystalline TiO_2_ surface and a PCE of 6.54% was achieved in perovskite solar cells.^38^ In 2012, Kim et al. added a hole‐conductor, made of spiro‐OMeTAD, to a submicrometer‐thick mesoscopic TiO_2_ film deposited with (CH_3_NH_3_)PbI_3_ nanoparticles as light harvesters and reported a perovskite solar cell with a PCE of 9.7%.^39^ In the same year, following identical strategy, Lee et al. explored both a n‐type TiO_2_ layer and insulating Al_2_O_3_ scaffold to form meso‐superstructured perovskite solar cells and reached a PCE up to 10.9%.^40^ Since then, following these pioneer works, there were intensive researches focusing on organic–inorganic hybrid perovskite solar cells. For example, the first hole‐conductor‐free mesoscopic MAPbI_3_ (CH_3_NH_3_PbI_3_) perovskite/TiO_2_ heterojunction solar cell was achieved with a PCE up to 7.3% by Etgar et al.^41^ In 2013, Heo et al. utilized a mesoporous TiO_2_ layer as the electron transporting layer and have achieved a PCE as high as 12.0% under one sun illumination.^42^ Following this work, Ball et al. improved the PCE to 12.9% by using CH_3_NH_3_PbI_3−_ *_x_*Cl*_x_* under low temperature processing.^43^ After that, a planar structured perovskite solar cell utilizing a vapor deposition method was developed by Liu et al. and achieved a PCE up to 15%.^44^ In 2014, the PCE of planar perovskite solar cells was boosted to nearly 20%.^45^ Jeon et al. also achieved a certified high efficiency perovskite solar cell up to 16.2% by controlling the component of perovskite thin film.^46^ Recently, the new record PCE hits 22.1%^47^ which was certified by National Renewable Energy Laboratory. The heated competition in PCE shows a rapid augmentation in the research of perovskite solar cells.

While increasingly more researchers focus on boosting the PCE of perovskite solar cells and increasing the stability of hybrid perovskite materials, it has been recognized that it is of equal importance to apply organic–inorganic hybrid perovskites to other types of optoelectronic devices, for instance, lasers, LEDs, and photodetectors. As shown by Xing et al., a solution processed CH_3_NH_3_PbI_3_ film has at least 100 nm electron–hole diffusion lengths.^48^ Stranks et al. found that the diffusion lengths of charge carriers can be greater than one micrometer in the mixed halide perovskite.^49^ Further researches on charge‐carrier dynamics showed that the organic–inorganic hybrid perovskite materials have a low nonradioactive recombination rate.^49^, ^50^ These excellent optoelectronic properties making them attractive candidates to realize optoelectronic devices beyond solar cells, such as LEDs,^51^, ^52^, ^53^, ^54^, ^55^, ^56^ lasers,^57^, ^58^, ^59^ and photodetectors.^60^, ^61^, ^62^, ^63^, ^64^

In this review, we first briefly discuss the synthetic methods of metal halide perovskites, including the solution process method, vacuum evaporation, vapor assisted solution process, and chemical vapor deposition (CVD). The recent advances of three types of optoelectronic devices, including LEDs, lasers and photodetectors, are then discussed. Finally, we discuss the challenges and prospectives for their future development.

## 2 Synthesis of Organic–Inorganic Hybrid Perovskite Thin Films

To fabricate high performance optoelectronic devices based on organic–inorganic hybrid perovskites, it is very important to deposit such absorber layers into a uniform film with full coverage in a controlled manner. It has been widely agreed that the device performance is highly dependent on the fabrication process of perovskites. Several methods have been developed for the deposition of organic–inorganic halide perovskite thin films.^44^, ^65^, ^66^, ^67^, ^68^, ^69^, ^70^, ^71^, ^72^, ^73^, ^74^, ^75^

So far, the solution processing and the vapor phase deposition methods are widely used in fabrication of perovskite films. Both of the two types of methods have produced high efficiency solar cells and other optoelectronic devices. These methods will be briefly discussed as follows.

### 2.1 Solution Deposition Method

**Figure 2:**
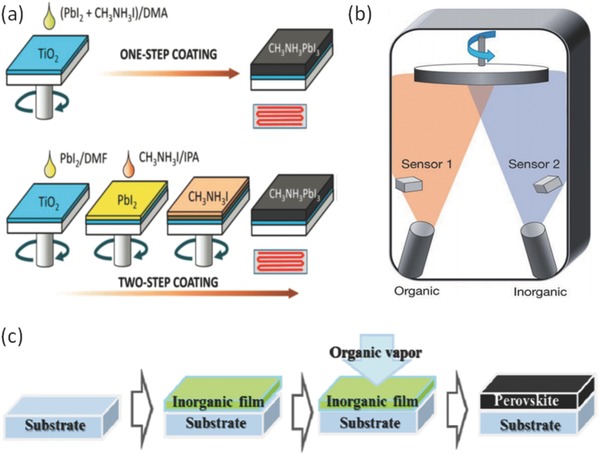
The schematic diagram of perovskite thin films deposition methods a) the solution processing methods with one‐step coating method and two‐step coating method. Reproduced with permission.^68^ Copyright 2014, AIP Publishing LLC. b) The vacuum vapor method. Reproduced with permission.^44^ Copyright 2014, Springer Nature. c) The vapor assisted solution method. Reproduced with permission.^84^ Copyright 2014, American Chemical Society.

In solution deposition method, the precursors were first mixed in a solvent in ambient. The thin films can be deposited by spinning coating, knife coating, spray coating, and/or printed methods. Solution deposition can be operated easily and show many advantages. In the early work, the perovskite thin films are mainly deposited by one step spin coating method (also known as one‐step method)^73^, ^76^, ^77^, ^78^, ^79^, ^80^, ^81^ as shown in **Figure** **2**a. However, the solution deposited thin films often rendered low coverage with small grain sizes and high surface roughness, which lead to the poor performance of the devices. In the one step method, mixed BX_2_ and AX precursors in a certain proportion are dissolved in a polar solvent such as *N*,*N*‐dimethylformamide (DMF) and dimethyl sulfoxide (DMSO). The mixed solvent is then deposited onto substrates by spin coating in order to rapidly evaporate solvent. During the evaporation of solvents, the perovskite crystallizes, finally rendering a perovskite thin film on the substrates. Note that as the solvent evaporates quickly, the crystallization of perovskite also becomes very fast, which renders low coverage of the substrate and hence a possibility of short circuits in devices. The “one step” method is difficult in controlling the quick crystallization of perovskite films during the solvent evaporation. Extensive pin–holes in perovskite thin films result in poor device performance and reproducibility.

In 2014, Seok et al. reported an improved method by mixing DMSO into the precursor solution followed by dripping antisolvents (e.g., methylbenzene) during the spin coating processing, which formed an intermediate perovskite phase on substrates.^46^ At 100 °C, the film of the intermediate phase is effectively changed into a crystalline perovskite thin film, which gives astounding control of film crystallization.

Burschka et al.^66^ proposed a modified method which is normally called two‐step method, as illustrated in Figure 2a, to improve the coverage of perovskite films. In this method, BX_2_ and AX are dissolved in different solvents. Lead iodide (PbI_2_) was then introduced into TiO_2_ nanopores by spin coating. The TiO_2_/PbI_2_ composite film was then dipped into a solution of CH_3_NH_3_I in 2‐propanol. Finally, a dark brown thin film of CH_3_NH_3_PbI_3_ was formed with good quality.

Xiao et al. also modified the two‐step spin coating method.^69^ First, they deposit a layer of PbI_2_ by spin coating and then followed by an annealing process. In contrast to the work by Burschka et al., instead of immersing the PbI_2_ film into the CH_3_NH_3_I solvent, they deposit CH_3_NH_3_I by spin coating, which leads to further improved film quality.

The processing method has been shown to play a key role in the perovskite film quality and corresponding device performance. Im et al. further compared the difference between the one‐step method and two‐step method. Films deposited by the one‐step method often have many pin holes which increase leakage current and provide a pathway for hole–electron recombination, and hence limits the device performance.^68^ Ko et al.^82^ also improved the two‐step method. The substrates were heated before spinning PbI_2_ solvent in order to obtain a highly infiltrated PbI_2_ thin film. In addition, Im et al. investigated the influence of CH_3_NH_3_I concentration on grain size.^83^

There are still many problems to be addressed in solution processing methods. First, two components were generally dissolved in the same solvent for the one step solution method. Therefore, a suitable solvent for dissolving both components is hard to find. The two components also have a high reaction rate. As a result, this method often renders a thin film with a high density of pinholes and incomplete coverage. Nonetheless, the solution processing methods are facile and flexible in preparing high quality perovskite films. Increasingly more efforts are devoted into this technique to tackle the existing issues.

### 2.2 Vapor Deposition Method

Vapor deposition method, on the contrary, could form uniform and high coverage perovskite thin films. Comparing to solution methods, thin films deposited with vapor methods often show a uniform, smooth, and compact surface with few pin–holes, which in turn improves the crystalline of perovskite thin films and device performance as well.

Liu et al. introduced a vapor deposition method for the fabrication of perovskite films.^44^ In this method, CH_3_NH_3_I powder and PbCl_2_ powder are placed in two separated sources in a vacuum system. As shown in Figure 2b, there are two sources in the vacuum chamber and each source was monitored by a sensor in order to control the deposition rate of each component. By carefully controlling the temperatures of the sources, the deposition rate can be finely controlled, leading to an ultrasmooth and fully covered perovskite thin film. This work has paved a brand new way to deposit uniform perovskite thin films.

The vapor deposition method fabricates highly uniform perovskite thin films and leads to the reduction of pin holes compared with solution processed perovskite thin films. However, note that the vapor vacuum method needs to maintain vacuum environment during the film formation and this not only costs more energy but also incurs higher expenses. As a result, the demand for low energy consumption and cost saving is yet to be solved in future.

### 2.3 Vapor‐Assisted Solution Method

Taking the drawbacks of both of the solution deposition and vapor deposition methods into consideration, a hybrid method mixed called vapor assisted solution method was developed with both evaporation and spin‐coating processes. In 2014, Chen et al. reported the vapor assisted solution method.^84^ In their method, a PbI_2_ thin film was first deposited onto a substrate by spin coating and then converted it into CH_3_NH_3_I in vapor ambient in order to form a CH_3_NH_3_PbI_3_ thin film, as shown in Figure 2c. By avoiding the vacuum system in film formation processing, this method lowers the cost of film deposition compared with the vacuum deposition method.

**Figure 3:**
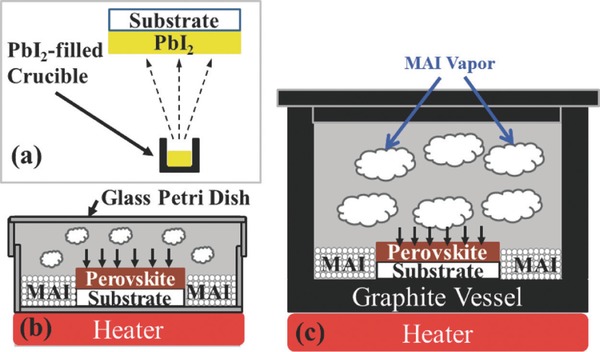
Three types of vapor deposition method compared by Abbas et al. a) PbI_2_ vapor evaporation b) with petri dish and c) with graphite vessel container. Reproduced with permission.^85^ Copyright 2015, AIP Publishing LLC.

In the hybrid method, the container plays an important role in thin film formation. Abbas et al. modified the vapor method with both a petri dish and a graphite container.^85^ The schematics of the experiment apparatus were shown in **Figure** **3**. In this study, the uniformity of thin film could be improved by the petri dish and the graphite container. Graphite is a conductor and eliminates the buildup of static charges during CH_3_NH_3_I deposition processing. This was believed to decrease the agglomeration of CH_3_NH_3_I into large particles.

To sum up, compared with solution processing methods, vapor methods often produce highly uniform and smooth thin films which have great advantages for device performance. On the other hand, solution processed perovskite thin films can be deposited at room temperature without any vacuum system, which have potential for low cost manufacturing.

### 2.4 CVD Method

There are many attempts to develop a new method that is suitable for large‐area and low‐cost fabrication of perovskite films. Apart from vapor assisted solution processes, CVD is another potential method.

Leyden et al. introduced CVD formamidinium based perovskite layer and achieved to fabricated a large area perovskite solar cells module which shows CVD method have potential application of fabricating large area perovskite thin film.^86^ This is important for industrialization.

Luo et al. used a low pressure CVD method to fabricate perovskite thin films.^71^ This approach can effectively reduce the intercalating reaction rate that too rapid to control and easily overcome this blocking issue during the solution process. They also try to control morphology by CVD method.^87^ They carefully control the Cl element in the CVD system in order to obtain a high quality perovskite thin film. This method can be carried out in ambient condition which is a competitive method for perovskite thin film deposition.

Although the thin film prepared by vapor deposition methods generally exhibits smooth and fully covered perovskite films, the solution‐based methods are still believed to be a good approach to achieve low‐cost and large‐area perovskite thin films. So far, there are yet several challenges to be solved in future. The first one is to increase the stability of perovskite thin film. The second one is that the reproducibility in large scale and the degradation mechanisms of perovskites need to be investigated further in order to meet the demand of industry production.

## 3 Light‐Emitting Diodes (LEDs)

The organic electroluminescence materials were discovered by Pope et al. in 1960s.^88^ In 1990s, with the achievement of the conjugated polymer‐based LEDs and the fabrication of flexible LEDs by Gustafsson et al., it has appealed the new idea for the application of organic LED (OLED).^89^ At present, OLED was regarded as a promising display technology. As OLEDs have many advantages such as integrated, wide‐gamut, full‐color displays, the OLED technology is believed to have potential to replace the liquid crystal display in the future. At the same time, the organic–inorganic hybrid perovskite materials can provide a new chance for develop another efficiency LEDs devices.

For years, direct band gap inorganic semiconductor has been studied for high efficiency optoelectronic devices. On the other hand, the development of organic–inorganic hybrid perovskites have proved that they have shown many unique properties such as strong photoluminescent quantum yields, long range electron–hole diffusion lengths, and low nonradioactive recombination rate, making them potential application for light‐emitting devices.^90^, ^91^, ^92^ As the perovskite solar cells have made a rapid progress in last few years, such efforts inspire applications of perovskites in other optoelectronics devices.

The organic–inorganic hybrid perovskite LEDs have been reported by Era et al.^93^ The hybrid perovskite (C_6_H_5_C_2_H_4_NH_3_)_2_PbI_4_ was used as an emitter layer. This early device operated at liquid‐nitrogen temperature and was not suitable for any commercial applications.

In 2014, Tan et al.^51^ fabricated a bright LED based on organic–inorganic hybrid perovskite materials. By tuning the halide composition in perovskite, they obtained green and red electroluminescence diodes. In this work, the hybrid perovskite materials were coated on the indium tin oxide (ITO) coated glass, then poly(3,4‐ethylene dioxythiophene):poly(styrenesulfonate) (PEDOT:PSS) was utilized as hole injector. The device structure is ITO/PEDOT:PSS/CH_3_NH_3_PbBr_3_/F8/Ca/Ag and the CH_3_NH_3_PbI_3−_ *_x_*Cl*_x_* is sandwiched between two larger‐bandgap titanium dioxide (TiO_2_) and poly(9,9′‐dioctylfluorene) (F8) layers. With deep ionization potential and a shallow electron affinity, the F8 polymer layer can confine the electron and holes in perovskite layer which in turn increases the quantum efficiencies of LED devices.

**Figure 4:**
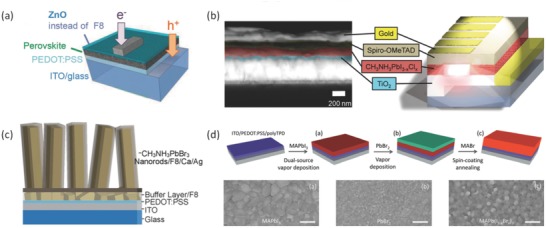
a) The perovskite LEDs (PeLEDs) structure with ZnO or TiO*_x_* instead of F8 as the electron injector. Reproduced with permission.^53^ Copyright 2015, John Wiley and Sons. b) Cross‐section scanning electron microscope (SEM) image of a device and the device structure showing the different layers. Reproduced with permission.^54^ Copyright 2015, American Chemical Society. c) Device structure of a CH_3_NH_3_PbBr_3_ nanorod array LED. Reproduced with permission.^55^ Copyright 2015, American Chemical Society. d) The processing method and surface morphology SEM image of MAPb(I_1−_ *_x_*Br*_x_*)_3_ thin film. Reproduced with permission.^94^ Copyright 2015, American Chemical Society.

Kim et al.^52^ reported another room temperature light‐emitting diode by using CH_3_NH_3_PbBr_3_ as an emitting layer and CH_3_NH_3_PbBr_3_ composed with PEDOT:PSS as so called self‐organized buffer hole injection layer (Buf‐HIL). The perovskite layer was deposited by spin coating. They achieved a bright and efficient perovskite LED. With the application of Buf‐HIL, the exciton quenching can be prevented and the hole injection can be increased. Those two features is the key point of forming bright perovskite LEDs. It is worth mentioning that no fluorene‐based layers were used as the electron barrier layer in the research of Hoye et al.^53^ As a result, they deposited a ZnO film onto green‐emitting methylammonium lead tribromide perovskite at only 60 °C, which is very competitive and economical as a low‐cost fabricating method. The structure of the LED is ITO/PEDOT:PSS/CH_3_NH_3_PbBr_3_/ZnO/Ca/Ag, as shown in **Figure** **4**a.

Jaramillo‐Quintero et al.^54^ showed another approach to achieve bright LEDs based on organic–inorganic hybrid perovskite materials. The device was fabricated by a solution process. The device structure is illustrated in Figure 4b, the electron injection layer and hole blocking layer is a thin TiO_2_ film, which is deposited by spray pyrolysis. After spin‐coating CH_3_NH_3_PbI_3−_ *_x_*Cl*_x_* layer onto the TiO_2_ layer, a spin‐coated Spiro‐OMeTAD layer is then deposited as a hole‐injection layer. The normalized photoluminescence (PL) spectrum showed that most of the light emits in near‐infrared region and a strong PL signal showed a low nonradiative recombination rate. The band structure, in which the TiO_2_ conduction band is closely below the conduction band of perovskite and the valance band of perovskite is slightly above the valance band of Spiro‐OMeTAD, is rendering an efficient electron and hole injection into the perovskite layer, which in turn leads to efficient light emitting.

Some specific nanostructures can also result in a promising performance of LEDs. As an example, a vertically oriented nanorod array enhances the active surface area and carrier injection efficiency. Wong et al.^55^ have achieved the first nanorod CH_3_NH_3_PbX_3_ light emitting diode. The perovskite nanorods were synthesized by a solution process. The poly(9,9′‐dioctylfluorene) (F8)/Ca layers and PEDOT:PSS were used to be electron and hole injection layers respectively as shown in Figure 4c. Compared to the perovskite thin film that shows poor coverage and provides shunt path, the nanorod LED showed higher efficiency.

Gil‐Escrig et al.^94^ attempted to fabricate a perovskite layer based on dual‐source vapor deposition and utilized it in LED application. The substrates were first exposed under vapor CH_3_NH_3_I followed by PbI_2_ vapor in a vacuum chamber. Afterward, the MAPbI_3_ perovskite can be deposited onto substrates. After formation of the MAPbI_3_ film, the substrates were exposed under PbBr_2_ ambient, followed by spin coating MABr in isopropanol. In this study, the perovskite active layer was sandwiched between a poly(N,N′‐bis(4‐butylphenyl)‐N,N′‐bis(phenyl)‐benzidine) (polyTPD) electron emitting layer and [6,6]‐phenyl‐C61‐butyric acid methyl ester (PCBM) hole emitting layer. The vacuum‐based processes can tune the bandgap of perovskite by controlling the ratio of iodide/bromide and obtain a perovskite material with bandgap of 1.70 eV, leading to bright LEDs, as shown in Figure 4d.^94^

So far the solution‐based organic–inorganic hybrid perovskite LEDs have been developing rapidly. There are still room for improving the lifespan and high power LED devices. It is worth to mention that the efficiency and the lifespan of LEDs are also affected by the structure of the devices apart from the materials. The sandwich structures are usually studied for perovskite LED, and however, it may be useful to apply multiple layers and insert doping layers to optimize the preference of devices and spectrum selectivity.

Given the instability of organic–inorganic hybrid perovskite materials, there are many strategies developed to improve the stability of perovskite LEDs. Some polymers have been applied for protecting perovskite layers, while it is still not enough for reaching the performance of commercialized LEDs. More efforts should be devoted to improving the stability of perovskite LEDs. Furthermore, Pb is widely used in perovskite LEDs, such toxic elements impeded the further application of perovskite LEDs. Recently, lead‐free hybrid perovskite materials which replace Pb with Sn have paved the way for future hybrid perovskite LEDs. There are still high demands for achieving stable, efficient, high‐speed, bright, and large‐area perovskite LEDs as a low‐cost alternative technique for numerous applications.

## 4 Lasers

Since the first invention of ruby‐laser (Cr:Al_2_O_3_) by Maiman in 1960^95^ and later neodymium‐doped yttrium aluminium garnet (Nd:YAG) lasers in 1970s, solid‐state lasers have become a key technology for communication, materials fabrication, medical care, and optical imaging processing. To date, a rich variety of materials have been applied in lasers with emission from the ultraviolet to near‐infrared region such as ZnO, GaN, CdS, and GaAs.^96^

In recent years, organic–inorganic metal halide perovskite materials have been proven to be a promising optical gain material for lasers. Meanwhile organic–inorganic hybrid perovskite can be fully deposited by simple solution‐based methods and particularly, single crystal organic–inorganic hybrid perovskite can be acquired by solution growth. Recently, several works have reported on the fabrication of room‐temperature single crystal perovskite lasers.^57^, ^58^, ^59^, ^97^, ^98^ A recent work suggested that perovskite films can also possess excellent optical gain in a wide range of wavelengths.^99^

Xing et al.^100^ studied the intrinsic gain of perovskites by examining the amplified spontaneous emission (ASE) in a cavity‐free configuration. As a result of low bulk defeats density in CH_3_NH_3_PbI_3_ films of, the ASE threshold carrier density was calculated to be as low as ≈1.7 × 10^18^ cm^−3^, which means that this device can obtain adequate gain and results in an efficiency laser.

**Figure 5:**
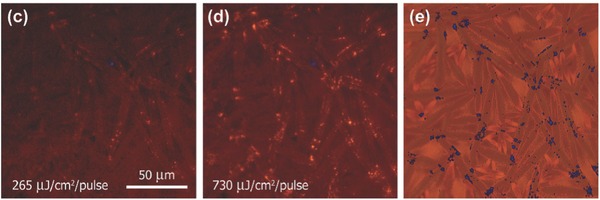
Micro‐PL images showing the spatial distribution of emission at the pump intensities. Reproduced with permission.^101^ Copyright 2014, AIP Publishing LLC.

Random lasing in planer MAPbI_3_ perovskite was achieved by Dhanker et al.^101^ They found low lasing thresholds <200 µJ cm^−2^ per pulse and narrow linewidth (Δλ < 0.5 nm). As shown in **Figure** **5**, the bright spot in (c) and (d) is where laser light is efficiently outcoupled from the perovskite network. The low‐threshold coherent random lasing shows that the lead halide perovskite can be used as gain materials.

Initially, the gain layer can be sandwitched between two top and bottom mirrors, Bragg reflectors, which simply form the vertical cavity surface emitting laser (VCSEL).^102^, ^103^ Compared to the VCSEL, which can be obtained easily, whispering‐gallery mode (WGM) microdisk lasers (MDLs) utilize successive total internal reflection along the disk circumference and provide high cavity quality factor (*Q*) and small mode volume (*V*) for integration of miniaturized devices.^104^

**Figure 6:**
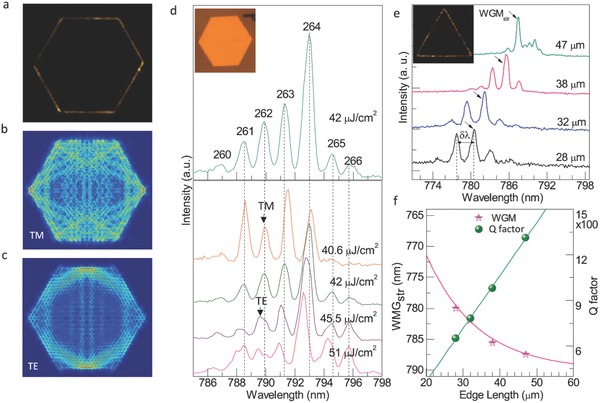
Whispering‐gallery mode analysis of the perovskite nanoplatelet laser. a) Far‐field lasing image, simulated field distributions at resonant cavity mode: b) transverse magnetic mode and c) transverse electric mode of typical hexagonal CH_3_NH_3_PbI_3_ nanoplatelets. d) Lasing spectra of hexagonal CH_3_NH_3_PbI_3_ nanoplatelets e) Lasing spectra is dependent on the edge length of a triangular CH_3_NH_3_PbI_3_ whispering‐gallery cavity. f) The wavelength of lasing modes (pink star dots) and Q‐factor (dark yellow dots). Reproduced with permission.^105^ Copyright 2014, American Chemical Society.

One of the NIR solid‐state nanolasers based on organic−inorganic perovskite CH_3_NH_3_PbI_3−_ *_x_*X*_x_* (X = I, Br, Cl) nanoplatelets were studied by Zhang et al.^105^ They used organic−inorganic lead halide perovskite nanoplatelets as microdisks that support WGM. The WGM can be observed in perovskite nanoplatelets as illustrated in **Figure** **6**. These devices show large exciton binding energy, long diffusion length, as well as promising quantum yields lead to strong, stable, and well‐controlled lasing actions. This was a good example for showing the organic–inorganic hybrid perovskite as a good candidate of WGM lasers.

By using a facile one‐step solution self‐assembly method, Liao et al. synthesized the CH_3_NH_3_PbBr_3_ MDs and WGM modes were achieved.^58^ What is more, perovskite MDLs can tune the wavelength by replacement of Br^−^ and Cl^−^ in green range from 525 to 557 nm.

**Figure 7:**
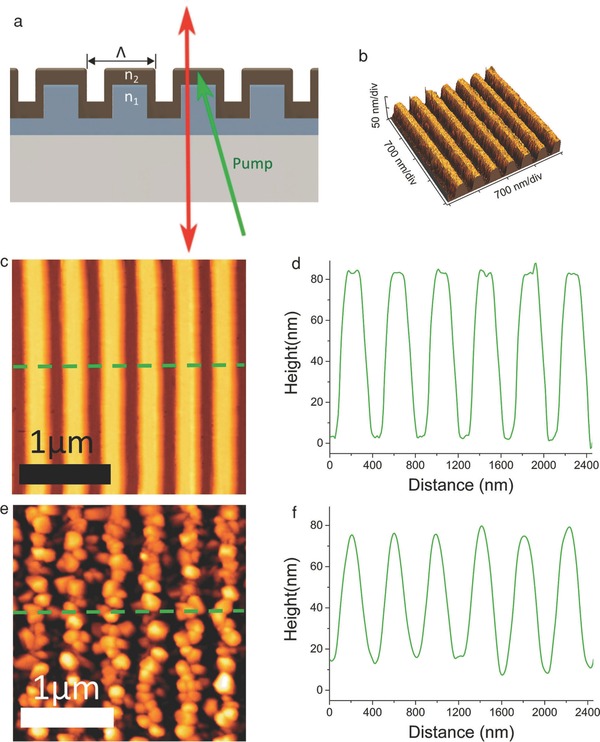
Schematic diagram of a generic distributed feedback (DFB) cavity. Reproduced with permission.^106^ Copyright 2016, John Wiley and Sons.

The distributed feedback (DFB) cavity structure was achieved by Saliba et al. as shown in **Figure** **7**.^106^ The research of distributed feedback cavity structure was pioneered by Kogelnik et al. in 1971.^107^ DFB perovskite cavities have huge potential as inexpensive, mirror free, widely tunable, and single mode lasers that are easy to manufacture in a large scale. The DFB structure is highly versatile and can be optimized, for example, toward lower thresholds or different output energies. Broad tunability may be achieved by utilizing the various organic–inorganic perovskite and tailoring the cavity to the gain maximum of the unpatterned film.

Nanowire structure has been applied in solar cells for a long time.^108^ Nanowire lasers are also believed to achieve high gain and strong confinement of photonic modes guided along the axial direction. Zhu et al. reported a lead halide perovskite nanowire laser with lasing thresholds of 220 nJ cm^−2^ and high quality factor up to around 3600.^57^ Fu et al. improved the synthesis of high‐quality single crystal perovskite nanowires, and successfully attain formamidinium lead iodide perovskite (FAPbI_3_), MABr‐stabilized FAPbI_3_, FAPbBr_3_, (FA,MA)PbI_3_ alloys, and (FA,MA)Pb(Br, I)_3_ double alloys, with their work, several types of FA‐based nanowire perovskite lasers were compared together.^59^ In general, the hydrogen bonding between FA cations will increase the stability of FA‐based perovskite materials. And as there are more possible perovskite materials are synthesized, more emission spectra can be covered with the FA‐based nanowire perovskite laser. This work shows that solution processed lead halide perovskite materials with its unique optical properties have great potential to be applied in lasers.

Meanwhile, the stability of perovskite also influences the devices performance and many modification methods were applied in improving the stability of perovskite lasers. A water‐resistant perovskite polygonal microdisk laser was fabricated by Zhang et al.^109^ By embedding water‐resistant polymer thin‐film with high flexibility and transmission onto polygonal microdisks, this structure was an excellent built‐in WGM microresonator for lasing. The polymer can prevent water and moisture from perovskite microcrystalline for improving the water resistance of the devices.

Aiming to increasing the quality factor, nanostructured hybrid perovskites have been also investigated, for example, perovskite microcrystal laser,^110^ perovskite microrod laser^111^ and perovskite quantum dot LEDs have been obtained^112^ and it is a good example for the chance of applying perovskite quantum dots in lasers. As discussed above, the organic–inorganic hybrid perovskite laser can be realized by different cavity configurations and it is important to improve the stability of perovskite materials and progressing on patterned technology in order to achieve better performance of corresponding laser. The perovskite laser devices can be applied in many fields like display, sensor, and lab‐on‐chip system in the future.

## 5 Photodetectors

Photodetectors are important optoelectronic devices for imaging, communication, automatic control, and biomedical sensing. The organic–inorganic hybrid perovskite materials have potential abilities to sense the spectra from visible to NIR, and even to X‐ray, which is very competitive to other material systems for photodetectors. Organic–inorganic hybrid perovskite photodetectors are mainly fabricated by solution processing methods^60^, ^61^, ^62^, ^113^, ^114^, ^115^, ^116^ owing to its low cost and suitability for room temperature processing. These features are very crucial for the fabrication of flexible photodetectors. Normally, organic–inorganic hybrid perovskite photodetectors are fabricated in a photoconductor, photodiode, or phototransistor structure.^117^, ^118^, ^119^, ^120^, ^121^, ^122^, ^123^, ^124^, ^125^

In order to decrease defects and grain boundaries in 3D bulk crystals, researchers attempted to synthesis 2D perovskite nanoflakes.^58^, ^126^, ^127^, ^128^ While synthesis of 2D perovskite faced many problems such as poor chemical stability, fast crystallization rate, and the intrinsically non‐van der Waals‐type 3D characteristics of perovskite, Liu et al. prepared 2D CH_3_NH_3_PbX_3_ perovskite as thin as a single unit cell through a combined solution process and vapor‐phase conversion method.^129^ In their study, the current can be enhanced significantly and high photo responsivities of 22 and 12 A W^−1^ were obtained with a voltage bias of 1 V. The excellent optical properties make the 2D perovskite a promising candidate for high‐performance photodetectors.

Hybrid perovskite single crystals are also ideal X‐ray and gamma‐ray detecting materials owing to their large mobilities, carrier lifetime and high atomic numbers of Pb, I, and Br. Wei et al. fabricated a single crystal hybrid perovskite X‐ray photodetector. Under continuum X‐ray energy up to 50 KeV, the detection efficiency was up to 16.4% at near zero bias which is four times higher than the sensitivity achieved with α‐Se X‐ray detectors.^130^ Recently, Wei et al. have developed a hybrid perovskite X‐ray photodetector. The assistance of brominated (3‐aminopropyl) triethoxysilane (APTES) molecules, which contact both perovskite and Si, led a significant reduction in dark current at higher bias, rendering a sensitivity of 2.1 × 10^4^ µC Gy_air_^−1^ cm^−2^ under 8 KeV X‐ray radiation.^131^

The first visible‐blind UV hybrid perovskite photodetector was realized by Adinolfi et al. Based on an MAPbCl_3_ single crystal, the detector achieved response times of 1 ms and showed a rising edge positioned at ≈420 nm.^132^ This shows that the organic–inorganic hybrid perovskite materials have excellent and adjustable spectra tunability.

In addition, some works are focusing on transition metal dichalcogenides which play an important role in optoelectronics.^133^ Based on a methyl‐ammonium lead halide perovskite (MAPbX_3_)/MoS_2_ hybrid structure with (3‐aminopropyl) triethoxysilane doping, another perovskite detector with high photoresponsivity (1.94 × 10^6^ A W^−1^) was fabricated by Kang et al.^134^

**Figure 8:**
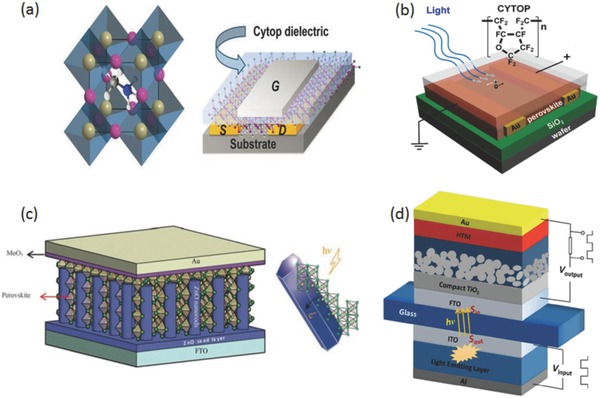
a) The schematic structure of the FETs on hybrid halide semiconductor layer, having Au source and drain contacts, Cytop dielectric, and Al gate electrode. Reproduced with permission.^135^ Copyright 2015, Cambridge University Press. b) The structure of photodetector with a layer of fluorous polymer coating which can increase the lifetime of the device. Reproduced with permission.^62^ Copyright 2015, American Chemical Society. c) Schematic diagram and energy level diagram of the as‐fabricated perovskite photodetector. Reproduced with permission.^137^ Copyright 2016, Published by The Royal Society of Chemistry. d) The schematic diagram of an organic–inorganic hybrid optocoupler. Reproduced with permission.^139^ Copyright 2015, Nature Publishing Group.

There are some examples^135^ that utilized FET which can be controlled by voltage between gate and source. A simple field‐effect phototransistor was achieved by Mei et al.^135^ In their research, the hybrid perovskite was the semiconductor layer and fluorous polymer (CYTOP) was the dielectric layer. Source and drain were Au electrodes while Al was used as the gate electrode, as illustrated in **Figure** **8**a.

The device shown in Figure 8b was fabricated by Guo et al. They discovered that photodetectors can exhibit a long‐time stability in air by spin coating a layer of water‐resistant CYTOP on the surface of CH_3_NH_3_PbI_3−_ *_x_*Cl*_x_*.^62^

A self‐powered structure represents a new type of photodetectors that can work without power supply. They can generate electric signal on their own. The first self‐powered photodetector based on organic–inorganic hybrid perovskite was carried out by Su et al.^136^ After that, Yu et al. applied ZnO nanorod/perovskite heterojunction self‐powered perovskite photodetector, as shown in Figure 8c.^137^

Maculan et al.^64^ found that the solubility of MAPbCl_3_ power in a mixture of DMSO‐DMF (1:1 v/v) decreased solubility at elevated temperature and the MAPbCl_3_ crystal was obtained by inverse temperature crystallization. Then the MAPbCl_3_ single crystal was utilized as the active layer in a photodetector. The device showed a high detectivity and high ON/OFF ratio. The response time was in the order of millisecond. Moreover, the MAPbCl_3_ single crystal exhibits long term stability in ambient condition. MAPbI_3_ single crystal was also applied in photodetectors by Ding et al.^138^ In their study, an asymmetric Au‐Ag electrode was employed and the photocurrent was almost two orders of magnitude larger than that based on a perovskite polycrystalline film with a similar device structure.

In addition, optocoupler is a common application of photodetector and plays an important role in communication, using photodetector as a part of an optocoupler was achieved by Li et al. as shown in Figure 8d. In this work, a tandem OLED was used as a light source.^139^ The photodetector was fabricated by organic–inorganic hybrid perovskite. The optocoupler exhibits rapid frequency‐response in microseconds, which shows that organic–inorganic hybrid perovskite materials are very promising candidates for practical use in optoelectronic devices.

The organic–inorganic hybrid perovskite photodetectors benefit from the tunable bandgap which can select the sensing spectrum of the devices. For photodetectors, it is important to control the roughness of perovskite thin film. As previously studied, the perovskite thin film deposited by vapor methods is better than deposited by solution methods, it is worthwhile to try vapor deposited perovskite thin films in photodetectors.

## 6 Conclusion and Perspectives

Besides the huge success in solar cells, organic–inorganic hybrid perovskite materials have also obtained a tremendous attention in the application of alternative optoelectronic devices such as LEDs, lasers, and photodetectors.

As we described before, organic–inorganic hybrid perovskite materials still face big challenges of poor long‐term stability, such as low thermal stability and humidity stability. This shortage is a bottleneck of the further applications of organic–inorganic perovskite materials. Another challenge is that some common hybrid perovskite materials contain heavy metals, like lead. It is necessary to develop lead‐free devices for environment friendly applications.

To conclude, this review presented a brief discussion of the progress and development in the field of organic–inorganic hybrid perovskite devices, especially the devices beyond solar cells. With the rapid evolution of perovskite solar cells in the last 5 years, the organic–inorganic hybrid perovskite materials have exhibited excellent optoelectronic properties, for example, long carrier diffusion lengths, high charge‐carrier mobility, low exciton binding energy, and bandgap tunability. As a result, devices, such as LEDs, lasers, and photodetectors, based on organic–inorganic hybrid perovskite materials have been successfully demonstrated.

The deposition methods from one‐step solution method to the novel CVD method have improved the surface‐coverage and the overall quality of perovskite thin films, which make fine control of the structural and electronic properties of perovskite films possible within the devices. The invention of perovskite LEDs opens new opportunities for cost‐effective and efficient light emitting devices. These works also inspire the application of organic–inorganic perovskite materials in lasers. Moreover, the superior optical properties and facile preparation methods of perovskite films also make them great alternatives for applications in photodetectors. Promising devices have been for photodetection from NIR to visible spectrum, and more recently, even for X‐ray and gamma rays.

As a potential candidate for future optoelectronic devices, organic–inorganic hybrid perovskite materials must overcome the obstacles on the way to massive industry production. For instance, perovskite materials must be deposited in large‐area while maintain their excellent properties. Also, perovskite materials have to be stable in long term in operation conditions and should even be able to tolerate harsh environments for broader applications. For these reasons, the research communities need to take further actions to address these issues, which could be development of new protection methods, such as encapsulation processes. Despite the challenges, organic inorganic metal halide perovskites offer promising alternatives for developing high performance and low cost optoelectronic devices. Given the great progresses made in such a short duration, and metal halide perovskite optoelectronics is expected to have a bright future.

## Conflict of Interest

The authors declare no conflict of interest.
